# Can Science-Based Targets Make the Private Sector Paris-Aligned? A Review of the Emerging Evidence

**DOI:** 10.1007/s40641-022-00182-w

**Published:** 2022-04-27

**Authors:** Anders Bjørn, Joachim Peter Tilsted, Amr Addas, Shannon M. Lloyd

**Affiliations:** 1 Department of Management, John Molson School of Business, Concordia University, 1450 Guy St, Montréal, QC H3H 0A1 Canada; 2 Department of Geography, Planning and Environment, Concordia University, 1455 de Maisonneuve Blvd. W, Montréal, QC H3G 1MB Canada; 3 Environmental and Energy Systems Studies, Department of Technology and Society, Lund University, Box 118, 221 00 Lund, Sweden; 4 Department of Finance, John Molson School of Business, Concordia University, 1450 Guy St, Montréal, QC H3H 0A1 Canada

**Keywords:** Climate change, Paris Agreement, Science-based targets, Corporate emissions, Literature review

## Abstract

**Purpose of Review:**

Companies increasingly set science-based targets (SBTs) for reducing greenhouse gas emissions. We review literature on SBTs to understand their potential for aligning corporate emissions with the temperature goal of the Paris Agreement.

**Recent Findings:**

SBT adoption by larger, more visible companies in high-income countries has accelerated. These companies tend to have a good prior reputation for managing climate impacts and most appear on track for meeting their scope 1 and 2 SBTs. More research is needed to distinguish between substantive and symbolic target-setting and understand how companies plan to achieve established SBTs. There is no consensus on whether current target-setting methods appropriately allocate emissions to individual companies or how much freedom companies should have in setting SBTs. Current emission accounting practices, target-setting methods, SBT governance, and insufficient transparency may allow companies to report some emission reductions that are not real and may result in insufficient collective emission reductions. Lower rates of SBT diffusion in low- and middle-income countries, in certain emission-intensive sectors, and by small- and medium-sized enterprises pose potential barriers for mainstreaming SBTs. While voluntary SBTs cannot substitute for more ambitious climate policy, it is unclear whether they delay or encourage policy needed for Paris alignment.

**Summary:**

We find evidence that SBT adoption corresponds to increased climate action. However, there is a need for further research from a diversity of approaches to better understand how SBTs may facilitate or hinder a just transition to low-carbon societies.

## Introduction

Science-based targets (SBTs) translate the temperature goal of the Paris Agreement to company-level greenhouse gas (GHG) emission reduction commitments. To date, more than 1000 companies have set SBTs, which are widely associated with serious intentions on climate action. For example, the UK government conflated SBTs with “strong climate credentials” in their call for corporate sponsors for the COP26 event [1], the Hague District Court referenced the SBT concept in their ruling ordering Shell to set more ambitious climate targets [2], the SBT concept inspired the European Commission’s climate benchmarks for Paris-aligned investments [3], and the Biden administration suggested that large federal suppliers be required to set SBTs [4].

The concept has also attracted critical attention. When the practice of setting SBTs was in its infancy, Trexler and Schendler [5] argued that SBTs could end up being an “unintentional, but devilishly clever way in which opponents of long-term climate action could help delay and undercut progress” by advancing “the mistaken notion that companies can substitute for public policy.” To this, Marland et al. [6] responded that the adoption of SBTs and other corporate climate initiatives “can be a positive force for the demand and implementation of climate policy.” Six years after this exchange, detailed guidelines for setting certified SBTs have been developed, data on companies’ engagement with SBTs have been generated, and a body of academic literature on the topic has emerged.

Here, we provide the first review of the academic literature on SBTs to shed light on their role in meeting the Paris temperature goal. We synthesize the findings of this literature using three lenses: existing company engagement with SBTs, appraisal of SBT methods and governance, and the prospects of SBT diffusion and interactions with policy. We then conclude with the implications of current findings and knowledge gaps for actors developing methods and guidance for setting SBTs, policymakers, and future research.

## Background: the Emergence of Corporate SBTs

### History

The SBT term has only recently been used in the context of companies^1^. Yet, the idea of linking corporate environmental performance and targets to external environmental goals is much older. For example, the Global Reporting Initiative has encouraged companies to disclose environmental performance in relation to “global limits on resource use and pollution levels” for two decades [7, 8]. A few companies followed suit early on [9, 10], but widespread adoption appears to have been hampered by a lack of external pressure and operational methods and tools, with some notable exceptions [11–13]. Instead, corporate emission targets largely appeared arbitrary, perhaps inspired by competitors’ targets, past performance, or what appeared achievable [14–17], and were often judged to lack ambition [18]. In 2015, the Paris Agreement rallied 196 nations “to limit global warming to well-below 2 °C above pre-industrial levels and pursue efforts to limit warming to 1.5 °C” [19]. The agreement’s polycentric governance approach [20] and the apparent insufficiency of national determined contributions to meeting its temperature goal [21, 22] meant that large companies, and other non-party stakeholders, came under pressure to take action [23, 24]. Around the same time, the Science-Based Targets initiative (SBTi) was created, coining the term “science-based target” in a corporate context, offering companies methods, tools, and guidelines for setting GHG emission targets aligned with the Paris temperature goal while acting as a target certifier [25, 26].

### The SBTi and Its Target-Setting Guidelines

SBTi is a partnership between CDP (formerly the Carbon Disclosure Project), the United Nations Global Compact, the World Resources Institute, and the World Wildlife Fund and is funded through target certification fees and various corporate and charitable foundations [25]. SBTi offers guidance to companies for calculating interim SBTs, which it evaluates and approves, and encourages companies with approved targets to disclose emissions and target progress annually [27].

SBTi currently recommends two target-setting methods, which are integrated in the organization’s target calculation tool, for scope 1 and 2 emissions^2^ [28]. The absolute contraction approach (ACA) [29] implies that all companies reduce absolute emissions by the same proportion. The sectoral decarbonization approach (SDA) [30] assumes that some sectors, based on the cost of mitigation, will reduce emissions faster than others while factoring in company-specific base year emission intensity and growth projections. While SBTi once allowed scope 1 and 2 SBTs to align with a 2 °C temperature goal, companies must now set SBTs aligned with a well-below 2 °C scenario or a more ambitious 1.5 °C scenario. Whereas ACA is broadly applicable, SDA is applicable to a handful of “homogenous” sectors and can currently only calculate SBTs aligned with 1.5 °C for the power sector.

For some sectors, SBTi allows or requires companies to use sector-specific target-setting methods, of which some are variants of ACA and SDA (e.g., for aviation [31]) and others take a distinctly different approach (e.g., for financial institutions [32]). For other sectors, dedicated methods and guidance are still under development. Importantly, companies in the emission-intensive oil and gas sector and the forest, land, and agriculture sector can commit to setting SBTs but must wait for sector-specific guidance in order to submit targets for validation [33, 34].

For scope 3 emissions^3^, SBTi’s target-setting requirements are less rigid, recognizing that companies are less able to accurately quantify and influence scope 3 emissions [27]. Companies must set SBTs for scope 3 emissions if they account for at least 40% of total scope 1, 2, and 3 emissions (which is often the case), and the targets must cover at least two-thirds of scope 3 emissions [27]. Companies can use target-setting methods other than the ACA and SDA methods (subject to certain constraints), and scope 3 targets are still allowed to align with a 2 °C scenario in addition to the well-below 2 °C or 1.5 °C scenarios. Companies may also set supplier or customer engagement targets instead of direct scope 3 emission reduction targets. With engagement targets, companies commit to motivating selected suppliers and customers to set SBTs for their scope 1 and 2 emissions.

All SBTs must cover a minimum of 5 years and a maximum of 15 years from the date of submission to SBTi for approval (complementary longer-term targets are also allowed). While companies are free to choose their base year, SBTi recommends the use of the latest year for which data without an atypical emission profile are available. In general, companies should express SBTs as percentage reduction in absolute emissions or emission intensities (e.g., per ton of product or $ of revenue). For scope 2, SBTi also permits targeting increased procurement of renewable electricity^4^. Scope 3 engagement targets involve targeting a percentage of suppliers and customers (by scope 3 emissions or procurement spend) with SBTs.

Whereas companies normally commit to setting an SBT, submit an SBT for approval, and wait for the SBTi to validate the SBT [27], the SBTi recently sets up a dedicated target approval route for small- and medium-sized enterprises (SMEs) (fewer than 500 employees) that bypasses these steps [35]. Instead, SMEs automatically have their targets approved when signing a letter committing to reducing absolute scope 1 and scope 2 GHG emissions 30% or 50% by 2030 from a 2018 base year (consistent with the ACA method for the well-below 2 °C and 1.5 °C goals, respectively) and to measure and reduce scope 3 emissions.

### Uptake of SBTs

**Fig. 1:**
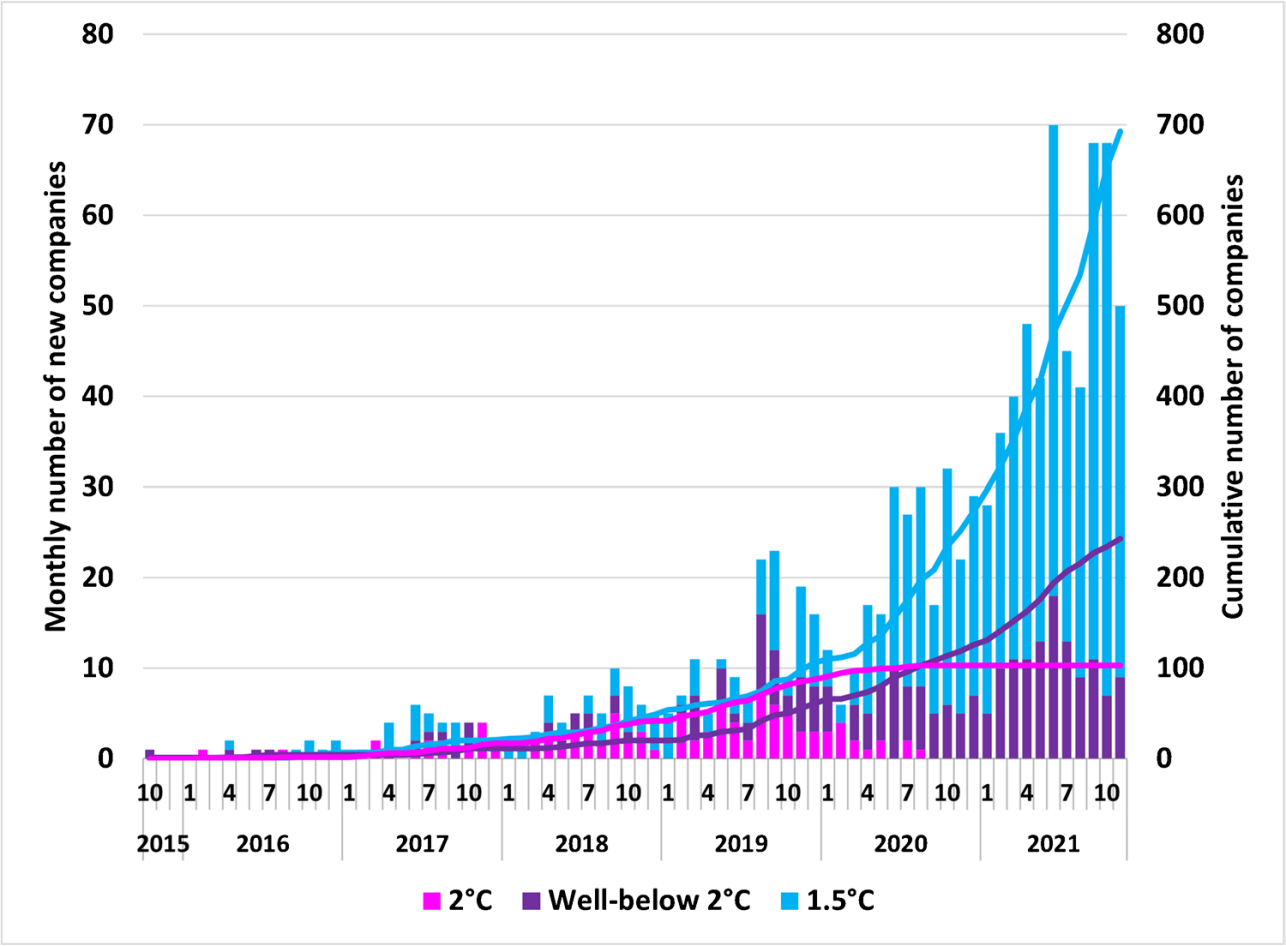
SBT approval for 1039 companies by temperature goal through November 2021. Data extracted from SBTi dataset on 2 December 2021 [37]. Companies who have only committed to setting SBTs are not included. Companies with revised targets are only counted at the timing of their latest target approval

Since its creation in 2015, SBTi has approved targets for more than 1,000 companies (Fig. 1). Each company typically has two or three individual SBTs that differ according to emission scopes covered and use of absolute or intensity-based targets and timeframe. As of October 2020, according to SBTi, companies with approved targets “were collectively responsible for 1.2 billion tonnes of scope 1 and scope 2 greenhouse gas emissions in their most recent reporting years, which is approximately 3.6% of global greenhouse gas emissions from energy and industry” [36]. Assuming a linear relationship between the number of companies with approved targets and share of global emissions suggests this has increased to 8.3% of global emissions (452 companies had SBTs in October 2020 and 1039 in November 2021). However, this may be an overestimate given the increase in SMEs with SBTs approved in 2021 (166 compared to 29 in 2020, two in 2019, and none in the preceding years). Company uptake was modest at first, but more companies had SBTs approved in the 12 months before November 2021 than the initial 62 months of the SBTi (Fig. 1). However, the recent rate of increase in SBT uptake shown in Fig. 1 is somewhat inflated because companies with revised targets are counted at the timing of their latest target approval^5^. In addition to companies with approved SBTs, more than 800 companies (not included in Fig. 1) have made commitments to setting an SBT in some (unspecified) future date. This indicates that the recent acceleration in target approval may continue. However, as of November 2021, 136 companies that announced commitments between 2015 and 2019 do not yet have an approved SBT^6^, indicating that not all commitments lead to approved SBTs. There has also been a shift in the temperature alignment of SBTs. While SBTs commonly aligned with a 2 °C scenario well into 2019, two-thirds of targets currently in place align with a more ambitious 1.5 °C scenario (Fig. 1).

**Fig. 2:**
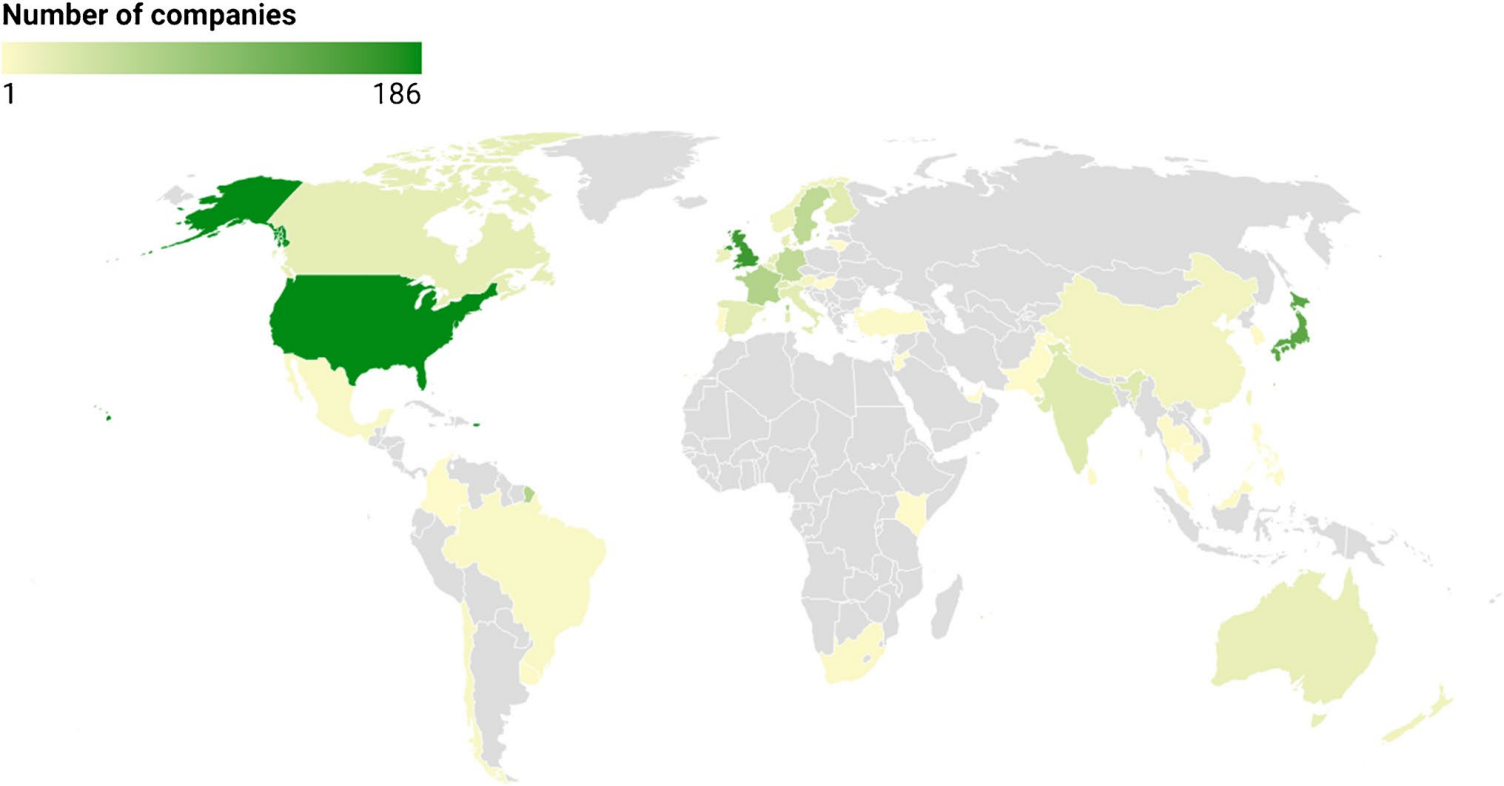
Geographical distribution of 1039 companies with approved SBTs through November 2021. Data extracted from SBTi dataset on 2 December 2021 [37]. Companies who have only committed to setting SBTs are not included. Two companies in Bermuda are not shown. Map created using Datawrapper [38]

**Fig. 3:**
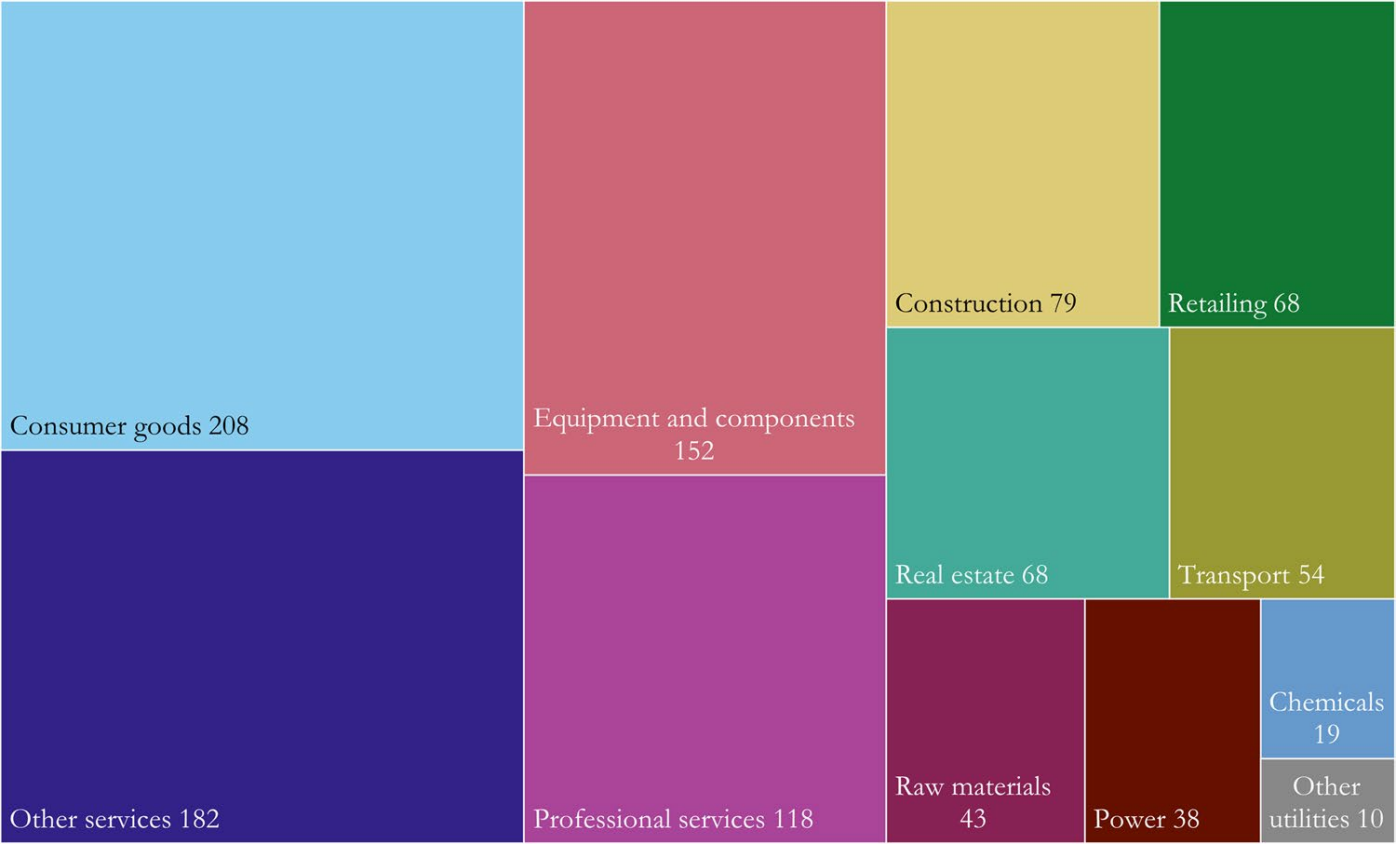
Sectoral distribution of 1039 companies with approved SBTs through November 2021. Data extracted from SBTi dataset on 2 December 2021 [37]. Companies who have only committed to setting SBTs are not included. See Table 2 in the Appendix for a translation between SBTi sectors and the sectors used here

As of November 2021, companies with headquarters in Europe account for more than half of approved SBTs, North American and Asian companies account for most of the remaining share, and companies from Latin America, Africa, and Oceania represent less than 6% of SBTs (Fig. 2). Companies with SBTs span a diverse range of industries and are frequently found within services, consumer goods, and equipment and components (Fig. 3). The low number of companies in the raw materials industry reflects the current lack of guidance for the oil and gas sector and the forest, land, and agriculture sector [33, 34].

### New Developments

SBTi is preparing a number of changes that may substantially alter the SBT landscape [39], including only approving SBTs that align with a 1.5 °C scenario from mid-2022 [40]; developing additional sector-specific methods and guidelines [34]; developing measurement, reporting, and verification guidance, which may make it easier for stakeholders to understand individual SBTs and companies’ progress against them [36]; and developing a standard for science-based net-zero targets^7^, which may contribute to much needed clarity and rigor to net-zero targets [41] and ensure both short-term and long-term action by building on SBTs [42]. Finally, the Science Based Targets Network (affiliated with SBTi) is developing methods and guidelines for setting SBTs for environmental issues other than climate change, such as land and water use [43].

## Methods: Identification of Literature

We considered literature already known to us, performed searches on Google Scholar^8^ and Web of Science^9^ on May, 25, 2021, and screened identified studies according to the following criteria:

- Must be published or submitted for publication^10^, in a peer-reviewed journal or conference proceeding or developed by a professional organization (e.g., excluding master theses)
- Must cover SBTs voluntarily set by companies (e.g., excluding country-level literature and literature applying the SBT concept for company or portfolio benchmarking)
- Must focus on SBTs for climate change (e.g., excluding literature focusing on water use)
- Must address SBTs substantially (e.g., excluding literature making brief references to SBTs as an example of a broader theme)
- Must involve a novel analysis (e.g., excluding SBTi guidance, SBT method documentation, and consultant reports on SBT-setting)

The process led to the identification of seventeen studies included in this review [5, 6, 26, 36, 44–53, 56–58]. Although our literature search was limited to two databases and potentially missed some relevant studies, particularly if not published in peer review journals, we believe it led to a solid basis for the review.

The first author reviewed all studies, identified relevant topics covered, and assigned a set of topics and associated studies to each author. Each author then evaluated the methods and findings of their assigned studies, performed an initial synthesis of the literature for their assigned topics, and presented their evaluation and synthesis to the full research team. The full research team collaboratively judged the interpretation and relevance of each study and synthesized the collective findings. Our findings are organized by the following research lenses, which emerged from this process:

1. *Existing company engagement with SBTs*: what drives companies to set SBTs, how does SBT adoption affect company climate action, and are companies on track to achieve their SBTs?
2. *Appraisal of SBT methods and governance*: do target-setting methods and governance align collective corporate action to the Paris temperature goal and is corporate emission disclosure sufficient for assessing this alignment?
3. *Prospects of SBT diffusion:* will voluntary SBT adoption continue to grow, will it be sufficient for aligning the private sector to the Paris Agreement, and how might SBTs interact with existing and emerging policy?

## Results and Discussion

The seventeen studies were mostly published in peer-reviewed journals, commonly related to the broad fields of climate change, environmental protection, or sustainability, but in some cases with a narrower focus, e.g., sustainable finance [56], global policy [45], and energy and buildings [47]. Half of the studies were published since 2020 [48, 49, 56–58] or were still undergoing peer review at the time of writing [51–53]. This indicates that academia is just beginning to study SBTs and data for empirical analysis is just beginning to emerge. The studies employ a variety of methods, such as statistical analyses informed by theories of corporate climate behavior (e.g., [52, 53, 57]), case studies of selected companies (e.g., [50, 51]), and reviews of SBT methods or SBTi guidelines (e.g., [26, 44, 49]), with many studies employing a mix of methods. Some studies focus on companies in specific regions (Europe, Asia, the UK, and Japan [50, 51, 56, 58]), or sectors (construction [47]), while most have a global scope. Some studies address SBTs as an element of a broader inquiry (e.g., [44, 52]), but we focus on their SBT content here. A summary of each of the seventeen studies is provided in Table 1. While our review focuses on these seventeen studies, we also draw on other literature when helpful in understanding SBT methods and practices.

**Table 1:** Summary of the seventeen reviewed studies chronologically ordered. Only the studies’ aspects related to SBTs are included. The lens numbers refer to existing corporate engagement (1), methods and governance (2), and diffusion (3)

Study reference	Lenses addressed	Methodological approach	Main outcome
Trexler and Schendler [5], 2015	2, 3	Commentary	Argues that SBTs hinder substantial emissions reductions by implying that corporate action can substitute for public policy, that decarbonizations is unrealistic without carbon pricing, and that only a few companies can reach their targets without ambitious regulation
Marland et al. [6], 2015	3	Comment on Trexler and Schendler [5]	Argues that SBTs and other corporate climate initiatives can be a positive force for the demand and implementation of climate policy
Giesekam et al. [47], 2018	2, 3	Review of SBT methods and SBTi material	Identifies several challenges for setting SBTs for the construction sector and problematizes the missing link between SBTs and national climate targets
Lister [45], 2018	2, 3	Review of SBT methods and SBTi material and the broader literature on corporate carbon management	Argues that lack of standardization in SBT-setting makes comparison of company targets impossible and proposes state co-regulation
Aden [44], 2018	2	Review of SBT methods and SBTi material	Provides a brief overview of seven SBT methods and outlines new research questions
Li et al. [46], 2019	2	Coupling of multiregional input − output database with existing SBT method	Presents a new method for setting SBTs for scope 3 emissions that is consistent with the sectoral decarbonization approach (SDA) [30] and adds sectoral and regional resolution
Faria and Labutong [26], 2019	2	Review of SBT methods and SBTi material and simulation of target-setting	Provides an in-depth description of four SBT methods and finds that SBTs can be as sensitivity to the choice of emission scenario as the choice target equation
Dagnet et al. [50], 2019	2, 3	Review of SBTs and corporate climate disclosure in the context of potential interactions with policy	Argues that the private sector and governments may form positive feedback loops related to data and climate ambition and discusses how this can take place in the context of SBTs, drawing on examples from Asia
Walenta [48], 2020	2, 3	Review of SBT methods and SBTi material and the broader literature on climate capitalism	Highlights a need for future critical research on the origins, diffusion and impacts of SBTs, the lack of uptake in some sectors, and the use of renewable energy certificates
Tuhkanen et al. [56], 2020	1	Review of corporate climate disclosure and documentations of green bonds	Investigates whether capital raised via green bond issuance is directed towards emission reduction targets but finds inconclusive evidence
Bjørn et al. [49], 2021	2	Review of SBT methods and SBTi material and simulation of target-setting	Provides an in-depth description of seven SBT methods and finds that individual methods and mixes of methods lead to emission imbalances (see footnote 17) of varying sizes and signs
Giesekam et al. [57], 2021	1, 2	Review of SBTs and corporate emission disclosure	Finds that most companies are on track to meeting SBTs for scope 1 and 2 emissions, but not for scope 3 emissions, and that insufficient emission disclosure prevents a complete picture of target progress
SBTi [36], 2021	1, 2, 3	Review of SBTs and corporate emission disclosure and literature on diffusion of innovations	Finds that companies with SBTs in combination have reduced scope 1 and 2 emissions at a rate exceeding what is required globally for meeting the 1.5 °C target, calls for higher quality in corporate emission disclosure. and proposes that reaching a threshold of 20% of companies with SBTs in a geography or sector will lead to rapid adoption by the remaining companies
Kuo and Chang [58], 2021	1	Ordinal logistic regression on the relationship between climate disclosure to CDP and CDP score for 1,994 Japanese companies	Determines that for companies in non-high-carbon emitting industries, there was a strong relationship between SBT adoption and the CDP score, and that in high-carbon emitting industries, the CDP score was higher for companies that adopted both SBTs and internal carbon pricing
Freiberg et al. [53], in review	1	Empirical analysis of the determinants of external standard (SBT) adoption and difference-in-differences research design assessing the impact of SBT adoption on climate efforts	Finds that companies are more likely to set SBTs if they achieved ambitious targets in the past, perceive economic risks to their business from climate change, and have carbon-intensive operations, finds that companies set more challenging targets and invest more in emission reduction initiatives after SBT adoption, and suggests that companies set SBTs to ensure sufficiently ambitious targets
Bolton and Kacperczyk [52], in review	1, 3	Statistical analysis evaluating the association of company, industry, and country-level characteristics with target-setting and the effect of target-setting on overall emissions	Finds that companies are more likely to set SBTs and other targets if they are larger, more visible, already disclose emissions, and have lower absolute emissions, finds a small but statistically insignificant relationship between the growth in target-setting and the reduction in annual growth of aggregate scope 1 emissions, concludes that target-setting initiatives have successfully drawn in companies most able and willing to commit but not companies that “that need to reduce their emissions the most”
Piper and Longhurst [51], in review	1	A sequential mixed methods approach, including literature review, discussions with “sustainability thought leaders,” surveys, and interviews to assess corporate action towards carbon neutrality among a small number of UK corporations	Finds that economics (rather than climate change concerns) is the main driver for target-setting and identifies credibility and standardization as the two predominant motivations for setting SBTs

### Lens 1: Existing Company Engagements with SBTs

According to the literature reviewed, companies are more likely to set emission targets (SBTs and others) if they are larger, more visible, and already disclose emissions and have lower absolute emissions (holding all else equal). Of these target-setting companies, those with higher emissions, higher perceived business risk from climate change, experience setting and achieving ambitious targets, and higher reputation for managing climate impacts are more likely to set SBTs. Findings that companies set SBTs to ensure sufficiently ambitious targets, confer legitimacy on climate efforts, and mitigate business risk from climate change suggest both substantive and symbolic motives. There is mixed evidence as to whether SBTs lead to more ambitious targets, but companies with SBTs report higher investments in emission reduction initiatives. There is some evidence that climate-related investments may not be sufficiently leveraged to achieve SBTs. Nonetheless, companies are making good progress towards achieving their scope 1 and 2 SBTs. Achievement of scope 3 SBTs has proven more challenging, likely due to the lack of control companies have over these emissions.

#### Drivers for Setting SBTs

Two preprint studies empirically investigated target-setting practices. Bolton and Kacperczyk [52] considered all publicly traded companies, regardless of whether they had set emission reduction targets, while Freiberg et al. [53] considered companies with declared emission reduction targets. Both distinguished between SBTs and other targets. Freiberg et al. [53] referred to the latter as “non-science targets” set via internal standards (e.g., benchmarking against peers’ targets or past performance). We instead use “internal targets” as there are instances of companies setting targets ambitious enough to be considered science-based by SBTi, without seeking SBTi approval. Bolton and Kacperczyk [52] evaluated the association of company, industry, and country-level characteristics with a company’s decision to set targets (i.e., internal targets, SBT commitments, and approved SBTs). They used Trucost, CDP (relying on corporate self-disclosure), and SBTi data from 2005 to 2019 for 17,385 publicly traded companies from 66 countries representing roughly 99% of global market capitalization. Of these, 1,957 (11.3%) declared an internal target and 455 (2.6%) set or committed to setting an SBT. They found that companies are more likely to set targets if they have higher fixed assets, are part of MSCI world index^11^, have higher stock price volatility, already disclose emissions, are under greater analyst coverage, have more women board members, have more antitakeover protections, and receive more financial media coverage about controversial decisions. Companies are less likely to set targets if they have a larger board, a higher average board member tenure, more board directors with a finance background, and if they are from the most or least carbon-intensive industries. These results are similar (albeit less significant) for committed and approved SBTs as for internal targets^12^. Companies are more likely to set an SBT if they previously set an internal target. The relationship between target-setting and the level of emissions varies by emission scope. For example, companies with higher scope 2 emissions are more likely to set internal targets but less likely to set SBTs. Overall, however, companies with higher absolute emissions are less likely to set targets. Companies were slightly more likely to set targets when their home countries set Intended Nationally Determined Contributions (INDCs) in the lead up to the Paris Agreement than they were when their home countries established Nationally Determined Contribution (NDC) after the Paris Agreement. Based on this, Bolton and Kacperczyk [52, 54, 55,] suggested that companies may feel pressure to signal intent alongside their governments, but less pressure to make commitments once governments takes more concrete action. Surely, there is some relationship between corporate target setting and national policy, but this finding offers only weak evidence, given that almost all countries have NDCs and most of these already had INDCs.

Freiberg et al. [53] investigated the reasons for using an external standard (i.e., setting an SBT) rather than an internal standard (i.e., setting an internal target). They hypothesized that companies set SBTs as a symbolic act to confer legitimacy on existing efforts or as a substantive commitment to ensure their target is sufficiently ambitious. They investigated support for these motivations using CDP data for 1,752 global firms that declared targets between 2011 and 2019, of which 385 (22%) had set an SBT^13^. Companies that had set more difficult past targets and successfully achieved past targets were more likely to set an SBT, lending support to both symbolic and substantive adoption. Companies that have more carbon-intensive operations, perceive more imminent climate change risks to their business, and perceive a greater impact from climate change risks are also more likely to set an SBT, suggesting an economic interest in addressing climate change and lending further support to substantive adoption (according to Freiberg et al. [53]).

Several additional studies touched on the drivers of SBT adoption. Piper and Longhurst [51] (also a preprint) surveyed eight companies (representing 25% of UK companies with SBTs at the time) about their carbon management practices followed by interviews of five of these companies. Participants acknowledged target-setting decisions tend to be driven by economics (rather than climate change concerns) and identified both credibility and standardization as the two predominant motivations for setting SBTs. According to participants, SBTs aid in future-proofing, linking individual climate actions to coherent measures, developing a sustainable carbon trajectory, making comparisons to other companies, and protecting the company’s reputation. Kuo and Chang [58] considered the relationship of SBT adoption and internal carbon pricing on the carbon management reputation of Japanese companies, with CDP scores used as a proxy for the latter. For companies in low-carbon emitting industries, there was a stronger positive relationship between SBT adoption and CDP score. In high-carbon emitting industries, CDP score was higher for companies that adopted both SBTs and internal carbon pricing. Since the CDP scoring methodology awards point for SBT adoption [59] and companies that report to CDP are more likely to set SBTs [52], it is not surprising that companies with SBTs tend to have higher CDP scores than companies without. However, the relationship between SBT adoption and other types of corporate climate action evaluated by the CDP scoring methodology remains unexplored, e.g., if SBT adoption has positive “spillover” effects on other types of corporate climate action evaluated (such as board oversight of climate-related issues) or if companies that are already doing well across the items evaluated by the CDP scoring methodology are likely to adopt SBTs. Regardless, the correlation of SBT adoption and CDP scores provides additional (albeit limited) support for increased credibility as a motivator for SBT adoption. Giesekam et al. [57] also observed that a significant proportion of global companies setting SBTs had already earned a high CDP score, reinforcing the importance of past success in driving SBT adoption.

#### Effect of SBTs on Companies’ Climate Action

Three studies considered the effect of SBTs on corporate climate action [52, 53, 56]. Freiberg et al. [53] used a difference-in-differences approach to benchmark companies’ SBTs against their previous internal targets to assess whether SBT adoption leads to more ambitious targets. They found targeted percentage reduction in emissions to increase 21% to 25% on average after companies set an SBT, depending on target coverage (percent of emissions covered by the target). However, since Freiberg et al. [53] also found a strong positive correlation between targeted percentage reduction and timeframe (for all target-setting companies), it is not clear if the targeted reduction rate (reduction per year) increases after companies set an SBT. Given the voluntary nature of SBT adoption and the inability to use an experimental design that randomly assigns companies to internal target and SBT groups, the authors warn against concluding that SBT adoption causes increased ambition.

Freiberg et al. [53] further assessed whether companies with SBTs increase their investments in emission reduction initiatives. Companies with SBTs reported 113% higher investments as compared to companies with internal targets and 63% higher investments as compared to companies with similar exogenous covariates (e.g., past target ambition, past target achievement) and factor variables (e.g., sector, country) likely to influence SBT adoption, confirming that increased investments are not driven by differences between the two groups of companies. In contrast, companies that set more ambitious internal targets did not invest more than companies that set less ambitious internal targets, indicating that increased investment is attributable to SBT adoption rather than setting ambitious targets. We note, however, that the study did not assess whether the level and type of investments typically associated with SBTs is sufficient for subsequently meeting the SBTs (addressed below). Freiberg et al.’s [53] findings that SBT adoption is accompanied by increased target difficulty and higher investment further suggest a substantive rather than symbolic commitment.

A somewhat different finding was presented by Tuhkanen and Vulturius [56], who investigated whether the twenty largest European corporate green bond issuers leverage proceeds from green bonds to fund emission reduction initiatives linked to their emission targets. Six of these issuers had approved SBTs when the study was carried out. A positive finding would have supported the assertion made in SBTi’s [36] 2020 progress report that “on financial markets we are seeing a movement towards science-based targets being embedded into sustainability-linked bonds.” Instead, Tuhkanen and Vulturius [56] found limited explicit reporting linking green bond funding to meeting the SBTs of the issuers. Overall, this indicates little pressure on green bond issuers to use proceeds to achieve SBTs.

Bolton and Kacperczyk [52] also investigated how target-setting and emission reductions changed over time. Between 2011 and 2019, the average reported scope 1 target (including internal targets, SBT commitments, and approved SBTs) increased from an 18.5 to a 30.5% reduction, but the average target timeframe also increased from 5 to 11 years, indicating (as in Freiberg et al. [53]) a possible trade-off between targeted emission reduction and timeframe. They also found that companies tend to report a higher percentage emission reduction in the initial years after setting a target than in later years, with a higher initial reduction observed for companies setting internal targets than those committing to or setting an SBT. The difference between this finding and that of Freiberg et al. [53] (related to SBT impacts on investments) suggests that SBT commitments are less substantive than approved SBTs (since Bolton and Kacperczyk [52] group committed and approved SBTs together). Bolton and Kacperczyk [52] further found that companies with lower initial emissions tend to achieve larger percentage reductions and are more likely to further strengthen their commitments, which aligns with Freiberg et al.’s [53] finding that companies are more likely to set SBTs if they had set and achieved more ambitious internal targets.

Bolton and Kacperczyk [52] also compared the rate of target adoption (including internal targets, SBT commitments, and approved SBTs) to the annual growth rate in aggregate scope 1 emissions for publicly traded companies tracked by Trucost between 2012 and 2019. Despite an increase in the share of companies setting targets, aggregate emissions continued to grow. However, the rate at which they grew fell in North America and Europe but went up in Asia, even though there was an increase in the share of companies setting targets in all three regions. The differences between regions indicate that aggregate emissions are more influenced by other causal variables (e.g., policy, offshoring, stakeholder pressure) than they are by corporate target-setting.

#### Performance Against SBTs

Researchers have noted the difficulty in assessing SBT achievement given target years are still generally in the future and since SBTi does not prescribe emission pathways for companies to follow from base year to target year [53]. Two studies recently used corporate disclosures to evaluate company-level progress against an assumed linear reduction trajectory to determine if companies were ahead or behind on their SBT(s) or had already achieved it prior to the target year [36, 57]. Giesekam et al. [57] found that companies were behind on 35% of the targets, ahead on 44%, and had already achieved the remaining 21%. An analysis by SBTi [36] indicates^14^ that companies were behind on 42% of the targets, ahead on 49%, and had already achieved 9%. The reason the SBTi analysis suggests a substantially lower share of targets already achieved is likely because its sample included all companies (297) with targets approved prior to November 2020, whereas Giesekam et al. [57] only included (81) companies reporting at least 2 years of progress against their SBT as of February 2020. This sample difference may also explain why SBTi found a higher share of companies behind on their target(s), since more recent SBTs are typically aligned with a more ambitious temperature goal (see Fig. 1). Although SBTi [36] covered more recent SBTs than Giesekam et al. [57], it should be noted that 20 companies are covered by the latter that are not covered by the former, according to our count, indicating that the two studies did not rely on the same corporate disclosure data^15^. Giesekam et al. [57] found that companies were significantly more likely to be ahead on their SBTs for scope 1 and 2 emissions than for scope 3 emissions. This is supported by the results of SBTi [36]^16^, who also reported that its sample companies in combination reduced scope 1 and 2 emissions by 25% between 2015 and 2019, which exceeds the requirements of the absolute contraction approach for the 1.5 °C scenario [29]. Giesekam et al. [57] suggest that the poorer performance on scope 3 may be due to the fact that companies have less control over emissions for which they are only indirectly responsible. Giesekam et al. [57] also noted that for most of the SBTs already achieved, companies had made substantial progress prior to the year the targets were approved by the SBTi (but after the base year). Similarly, the SBTi dataset [36] shows that 6% of the SBTs that were approved in 2020 had already been achieved in that year, which was 5–10 years earlier than targeted. These findings beg the question to what extent emission reductions of companies with SBTs can be attributed to setting SBTs versus companies’ pre-existing course of action [57]. In addition, Bolton and Kacperczyk [52]’s observation that companies tend to reduce emissions at a higher rate in the initial years after setting a reduction target than in later years indicates that initial progress according to a linear reduction trajectory may not be followed by long-term target achievement.

### Lens 2: Appraisal of SBT Methods and Governance

Proposed methods for setting SBTs vary in their target equation, global emission scenario, and principles for allocating global allowable emissions to individual companies. Extant literature has focused primarily on the fairness and appropriateness of existing methods for setting SBTs and the extent to which they align corporate action with the Paris temperature goal. Even though SBTi recognizes two methods for setting SBTs, there is no academic consensus as to which criteria a method must meet, whether companies should have flexibility in selecting which method to use, and whether it is possible or desirable for current methods to be consistent with national commitments. There is concerning evidence that SBTi methods and emission accounting standards may not lead to the needed emission reductions, particularly related to the potential for misalignment between aggregate SBTs and global allowable emissions, the use of renewable energy certificates, and unresolved scope 3 accounting issues. More transparency from SBTi and companies with SBTs is needed to assess alignment of SBTs with the Paris temperature goal and increase the chance that SBTs will have the intended impact on global emissions.

#### Target-Setting Methods and Governance

Aden [44] provided a brief overview of the seven methods that the SBTi originally referred companies to (before it began recommending just the ACA and SDA), followed by more in-depth method characterizations by Faria and Labutong [26] and Bjørn et al. [49]. These studies establish that the seven SBT methods vary in their target equations and application of global emission scenarios linked to the Paris temperature goal. Across the seven methods, Bjørn et al. [49] identified six different principles for allocating global allowable emissions to individual companies, with each method reflecting up to four principles and all methods relying on the “Grandfathering” principle. Some allocation principles are defined by a method’s target equation, while others are defined by the adopted emission scenario [49]. In this regard, Faria and Labutong [26] show that SBTs can be as sensitive to the choice of emission scenario as the choice target equation. Note that the recent sector-specific methods [34] are not considered by these studies.

Bjørn et al. [49] did not directly address the fairness or appropriateness of the allocation principles they identified in the SBT methods but argued that the principles should be clearly communicated to companies and their stakeholders to facilitate understanding of the value judgment involved and informed method choices. Giesekam et al. [47] went further in arguing that the SDA method’s lack of attention to “common but differentiated responsibilities” between nations means that resulting SBTs are inconsistent with national commitments to the Paris Agreement, which tend to involve higher emission reductions for high-income countries than low- and middle-income countries [22]. Similarly, Aden [44] questioned how SBTs can “best address the equity and distributional challenges of common but differentiated responsibilities?” Finally, taking a fundamentally critical stance towards the very idea of allocating global allowable emission to companies, Trexler and Schendler [5] characterized the attempt to “draw an explicit link between individual corporate targets and achievement of a global emissions reduction goal” as “voodoo economics.”

Since the last couple of years, the SBTi has recommended only two of the original seven methods for scope 1 and 2 emission targets (ACA and SDA) [27]. Bjørn et al. [49] questioned the reasons for the method recommendation based on an initial analysis of emission imbalances^17^ that did not favor the two recommended methods. A broader question is whether companies should be allowed to choose any target-setting method that meets some (yet to be prescribed) criteria or if all companies should use the same method and who gets to decide these criteria or that method. Aden [44] considered the availability of multiple methods a strength, arguing that “there is not a single SBT method that is best in all sectors and company situations.” Similarly, Faria and Labutong [26] encourage practitioners to “consider the fitness of the method and scenario to the particular use case.” However, Bjørn et al. [49] showed that global allowable emissions will be substantially overshot if all companies choose the SBT method that result in the least challenging target, as also hypothesized by Freiberg et al. [53]. In recognizing this risk, SBTi recommends companies to “screen several of the methods and choose the method and target that best drives emissions reductions” [27]. However, the lack of a requirement to disclose the SBT method behind approved targets makes it difficult to know if companies follow this recommendation [49]. Against this backdrop, Lister [45] sees a need for the state to co-regulate SBTs in terms of establishing an SBT standard that defines a consistent methodology, involving a common base year and target year and alignment with national climate policy goals. This proposal addresses the concern of Giesekam et al. [57] which companies may deliberately choose base years that result in favorable targets and the observation of Giesekam et al. [47] that current SBT methods generally do not allow alignment with national climate goals. We note, however, that current national commitments in aggregate are insufficient for meeting the Paris temperature goal [22] (although decreasingly so with the pledges and initiatives announced at COP26 [60]). Therefore, alterations of SBT methods to align with national climate goals may defeat the purpose of SBTs. In addition, alignment with national targets may be impractical for multinational companies.

Regarding scope 3 targets, Li et al. [46] noted that the SDA method only covers a handful of globally aggregated sectors. In response, they used a global multiregional input − output database to translate the sectoral emission pathways used in the SDA method into geographically differentiated emission intensity trajectories (emissions per value added) for 57 economic sectors across 140 regions. This essentially allows companies to set scope 3 targets as a function of SDA-based scope 1 and 2 targets of individual scope 3 actors while accounting for regional differences in base year emission intensities. It is unclear to what extent the Li et al. [46] method has been adopted by companies. We note that a potential shortcoming of this approach is the underlying assumption that all companies in a given sector and region have the same average value chain, involving the same average emission intensities, regardless of whether a company has already taken efforts to source low-carbon products prior to setting an SBT.

#### Emission Disclosure in Relation to SBTs

In their abovementioned studies of company performance against established SBTs, SBTi [36] and Giesekam et al. [57] both reported issues with insufficient corporate emission disclosure, which prevented them from tracking performance against 49% (no data for 34% and poor data quality for 15%) and 21%, respectively, of initially considered SBTs. These discard rates indicate a need for standardized corporate disclosure on target progress, including for scope 3 engagement targets. In addition, Dagnet et al. [50] noted that the flexible SBTi guidelines mean that corporate emissions are not necessarily reported in a format that governments can easily use to inform policymaking. These issues relate to broader criticism of the largely self-reported, unaudited, and unverified nature of corporate emission data [61].

Even for companies disclosing emissions in a comprehensive and consistent way, there may be issues. Trexler and Schendler [5] expressed concern about companies making use of ineffective renewable energy certificates and carbon offsets in order to report emission reductions against their SBTs at a low cost, and Walenta [48] later presented similar concerns. SBTi explicitly prohibits the use of emission offsetting for reporting target progress [27], yet Giesekam et al. [57] observed this practice among companies with SBTs. SBTi does allow renewable energy certificates through the market-based scope 2 accounting approach of the Greenhouse Gas Protocol [62]. However, this practice should be scrutinized given evidence that corporate purchasing of renewable energy certificates often does not lead to additional renewable energy generation or actual emission reduction [63, 64]. Likewise, the language around SBTs for “renewable electricity procurement” is problematic, given that companies can meet such targets by purchasing renewable energy certificates, which do not involve any physical procurement of renewable electricity [65].

Specifically for scope 3 emissions, Li et al. [46] argued that it can be difficult for companies to obtain data from upstream and downstream actors (e.g., suppliers and customers), in accordance with the Greenhouse Gas Protocol standard that SBTi refers companies to [66], and that the different emission estimation methods allowed by the standard can lead to very different estimates. Scope 3 accounting issue have also been addressed by other scholars [67] and pose challenges for tracking progress against SBTs. For example, a company changing the method used to estimate (parts of) its scope 3 emissions can obfuscate whether an apparent emission decrease (or increase) is genuine or a model artifact. Changes to the scope 3 standard are likely needed to address some of these issues.

### Lens 3: Prospects of SBT Diffusion

While SBT uptake has increased substantially since the establishment of the SBTi in 2015, uneven representation of low- and middle-income countries and certain sectors poses a potential barrier for mainstreaming SBTs. Discussion about the role normative, mimetic, and coercive (regulatory) pressures might play in mainstreaming SBTs has been accompanied by descriptions of a few relevant initiatives (e.g., retailers engaging with suppliers on scope 3 emissions and government programs to support companies in setting SBTs), but systematic assessments of the effectiveness of such initiatives are lacking. There are debates about whether voluntary SBTs will enable more ambitious climate policy. Overall, the literature does not provide a compelling case that the voluntary approach will be sufficient for aligning the private sector to the Paris Agreement.

#### Mainstreaming SBTs

In 2015, Trexler and Schendler [5] hypothesized that only a few companies that “account for an infinitesimal share of global emissions” will consider setting SBTs. Six years later, that perspective is challenged by the number of companies setting SBTs, the rate of new adopters, and the combined emissions involved ([36] and Fig. 1). While it is unknown whether the number of companies with SBTs will continue to grow, some scholars argue for the existence of positive feedback mechanism in the uptake of voluntary climate initiatives. The SBTi refers to diffusion of innovations theory (referencing Rogers [68]), according to which “adoption of an innovation by 10–25% of a system’s members (i.e., the ‘critical mass’) is followed by rapid adoption by the remaining members” [36]. Likewise, Banda [69] argue that actors that have already joined private climate governance schemes can influence other actors to join through both market and normative power. However, Banda [69] also argue that a list of criteria must be fulfilled for a climate governance scheme to be effective, relating to integrity, uptake, ambition, resilience, transparency, and materiality. Government incentives may also increase the uptake of SBTs. In Japan, the ministry of the environment offers companies free advice from consultants on the setting of SBTs, which has facilitated high uptake in that country (Fig. 2), as recounted by Dagnet et al. [50].

SBTs are mostly set by companies in high-income countries, with mainstreaming in low- and middle-income countries seemingly far-off (Fig. 2). Yet, SBTs for scope 3 and the nature of global trade may offer a mechanism for increased uptake in lower-income regions. Dagnet et al. [50] present examples of a Western retailer (Walmart) and clothing brand (Levi’s) that have both engaged with suppliers in Asia as part of their scope 3 SBTs. Such engagements can result in suppliers committing to and, eventually, setting their own SBTs^18^. This highlights SBTs as a relevant research topic within the global value chain literature, which focuses on power asymmetries in inter-firm networks with multinational companies typically deciding the terms upon which inclusion into global value chains is negotiated [71–73].

The currently uneven representation of sectors among SBTs (Fig. 3 and [36]) also poses a potential barrier for mainstreaming. In this respect, Giesekam et al. [47] argued that the limited sectoral and technological resolution of emission scenarios applied for setting SBTs is problematic. The authors found that this particularly hampers the applicability of the SDA method to construction companies and other complex sectors that produce heterogenous, long-lived products that interact with other companies and actors. Likewise, Walenta [48] called for more attention to the lack of SBTs in oil and gas and agriculture.

#### Effects of SBTs on Climate Policy

Within the reviewed literature, a number of scholars share the position that SBTs (and private climate governance more generally) are not sufficient conditions for society-wide decarbonization in the scale that is needed [5, 6, 45]. For Trexler and Schendler [5], corporate SBTs represent a “costly distraction” [5] for aggressive global emission reductions by cloaking “ineffective actions as meaningful solutions.” The principal reasoning is that SBTs imply that corporate action can substitute for public climate policy since companies pledge to decarbonize even in the absence of carbon pricing and other regulation. In stark contrast, Marland et al. [6] see corporate action as a catalyst for change and argue for positive policy feedback effects arising from corporate initiatives such as setting SBTs. Premised on the continued failure of transnational and “top-down” climate governance and the need for a polycentric or “hybrid” approach, they see corporate efforts as an enabler and driver of more ambitious climate policy. Although stressing the importance of prescriptive policy, Lister [45] similarly states that corporate SBT-setting ideally “helps to fortify, translate and strengthen the international emission reduction goals.”

In their study of the drivers and effects of SBT-setting, Bolton and Kacperczyk [52] also explored two possible explanations for the formation of target-setting coalitions. In a “collective action” scenario, companies set targets and incur costs to reduce emissions with the expectation that other companies will follow, making it easier for governments to introduce market reforms, for which early SBT adopters will be better prepared. Alternatively, in a “best in class” scenario, companies with low emissions, with little difficulty reducing emissions, and already on a decarbonization pathway join CDP and SBTi to formalize and advertise their “best in class” status. According to the authors, their findings that target-setting has had little effect on overall corporate emissions and that national determined contributions (NDCs) do not necessarily correspond to increased target-setting support the “best in class” explanation rather than the positive policy feedback argument suggested in the “collective action” scenario. Regarding current SBT adopters, they conclude “unless their efforts are supported by public policy to curb emissions and institutional investor pressure, it will be increasingly difficult to persuade the vast majority of companies that are still on the sidelines to join the decarbonization commitment drive.” We note that recent increases in SBT uptake and policymakers’ references to SBTs (as mentioned in the introduction) could provide support to the “collective action” scenario.

Overall, the reviewed literature involves different assumptions and expectations of the “policy spill-over” [69] of SBTs. For example, Dagnet et al. [50] argue that SBTs, and the corporate emission disclosure involved, can inform policymaking, especially in low- and middle-income countries. Yet, questions of how and when SBTs may serve as drivers or barriers of more progressive and ambitious climate policy largely remain unanswered. For example, SBTi routinely encourages companies with SBTs to sign letters in support of more ambitious climate policies [70]^19^; all the while some companies with SBTs appear to be members of industry associations lobbying against such policies. From a broader perspective, Walenta [48] argues for the need for critical engagement with SBTs within the existing scholarship on the intersection of corporations and climate change and outlines a research agenda with the aim of understanding, among other things, the climate justice significance of SBTs.

## Implications for Stakeholders

Our literature review points to several actions that SBTi can take to improve the integrity of SBTs and the chances of a positive global impact. To increase transparency, SBTi could document the decision-making process and selection criteria behind its recommendation of two specific target-setting methods (ACA and SDA), explicitly communicate the value judgments embedded in target-setting methods, and require companies with approved targets to publish information about the target-setting method and related company data. To increase the legitimacy of SBTs, SBTi could require companies with approved targets to periodically report third-party verified emission (and activity) data to a central, open access database, prevent companies from reporting emission reductions that are not real through market-based mechanisms, and reconsider the concept of SBTs for “renewable electricity procurement.” To increase uptake and the potential for positive impact, SBTi should target companies not already recognized for their climate disclosures (e.g., by virtue of a high CDP score) and increase efforts to recruit SMEs, companies in low- and middle-income countries, and companies in high-emitting sectors.

Our study also points to implications for policymakers. First, voluntary SBTs cannot substitute for the policies needed to reduce GHG emissions sufficiently to meet the Paris temperature goal. The uptake across regions and sectors is too uneven and it is unlikely that initial target progress will continue in the absence of new policy. Moreover, the potentials for creative emission accounting and incomplete emission disclosure make it difficult to even assess target progress and the effect on global emissions. Second, if policymakers aim to co-regulate SBTs with the SBTi (as proposed by Lister [45]), work is needed to increase the transparency of target-setting methods and approved targets and ensure rigorous emission accounting (see above), provide regulatory backing to monitor compliance, and ensure enforcement and to consider (mis)alignments with national determined contributions (NDCs) and national net-zero targets. Third, given that most companies likely rely on new policies to meet their SBTs [52], policymakers could engage with companies and other relevant stakeholders about the design of such policies. This could further motivate uptake of SBTs and increase the likelihood of governments setting more ambitious national climate targets.

## Conclusions and Outlook

There is growing emphasis on the role of SBTs to decarbonize the private sector as part of global effort to achieve the temperature goal of the Paris Agreement. The emerging body of literature related to existing company engagement with SBTs (lens 1) shows a relationship between company engagement with SBTs and prior experience reporting emissions, setting internal targets and achieving emission reductions; SBT-setting companies on average are setting higher percentage emission reduction targets than internal target-setting companies (but perhaps over a longer timeframe), reporting increased investment towards emission reductions and making progress towards achieving scope 1 and 2 SBTs; evidence of both symbolic and substantive action; and lacking engagement from the largest emitting companies. Definitive conclusions are hindered by current incomplete and inconsistent corporate disclosures. Additional research is needed to understand the mechanisms linking emission reporting and internal target-setting and achievement to SBT setting, distinguish between symbolic and substantive SBT adoption, identify investments and actions that lead to SBT progress, assess companies’ ability to achieve SBTs in the long-term, understand the extent to which companies can influence and monitor scope 3 SBT achievement, and perhaps most importantly, understand the specific barriers to SBT adoption by high-emitting companies and companies in underrepresented regions. This will require access to standardized, transparent corporate disclosure on targets, investments, and progress as well as rigorous hypothesis formulation and testing to understand the specific drivers, barriers, and outcomes of SBT engagement. Moreover, there is a lot to gain from more diversity in methodological approaches including both qualitative (e.g., interview-based or ethnographic approaches) and mixed methods research.

At least seven methods for setting SBTs have been proposed, each using different target equations, global emission scenarios, and subjective allocation principles to translate global allowable emissions to individual companies. The emerging literature on target-setting methods and governance (lens 2) raises concerns as to whether the two recommended SBT-setting methods, use of renewable energy certificates, and flexible scope 3 accounting approaches and target-setting approaches will impede collective alignment with the Paris temperature goal. Apart from one study that theoretically validated the concern about existing methods leading to a combined overshoot of global allowable emissions, the validity and impact of these concerns remain largely untested. While these concerns can (and should) be theoretically and empirically analyzed, more fundamental questions relate to value judgments [74] inherently embedded in SBT-setting methods and governance, most notably with respect to the allocation of global allowable emissions to individual companies. More research is needed on the implications of value judgments embedded in SBT-setting methods, freedom of choice offered to companies during target-setting, and level of transparency required from companies about these choices and the and role of stakeholders in SBT governance and decision-making.

Regarding the prospects for SBT diffusion (lens 3), emerging literature shows some evidence of mainstreaming, but uptake is still low in the most polluting industries, in low- and middle-income countries, and by SMEs. Given the urgency of climate change, the insufficiency of current public policy, and the emphasis on voluntary SBTs as a means for decarbonizing the private sectors, there is a critical need for studies from a variety of perspectives on how SBTs interact with existing and emerging climate policy. This includes exploring under what conditions SBTs may speed up or slow down more ambitious policy and how SBT influence total corporate greenhouse gas emissions. Regarding the last point, SBTs should be positioned in the broader literature on political economy, political ecology, and climate justice by asking questions such as given the global nature of capital and commodity flows, how may SBTs facilitate or hinder a just development between the Global North and Global South? With its focus on production, how may SBTs impact (in)equalities in consumption and consumption-based GHG emissions? How do companies seek to square SBTs with continued profit accumulation and what are the consequences thereof? Addressing such questions reflects the reality that any operational target will always be a socio-political choice and highlights the need for continued reflexivity and critical scrutiny of SBT-related practices.

## Appendix

Table 2

**Table 2:** Translation from SBTi sectors to the sectors used in Fig. 3

SBTi sector	Sector used in Fig. 3	Number of approved SBTs through November 2021
Chemicals	Chemicals	19
Building products	Construction	20
Construction and engineering	Construction	33
Construction materials	Construction	17
Homebuilding	Construction	9
Consumer durables, household, and personal products	Consumer goods	54
Food and beverage processing	Consumer goods	86
Textiles, apparel, footwear, and luxury goods	Consumer goods	61
Tobacco	Consumer goods	7
Aerospace and defense	Equipment and components	1
Automobiles and components	Equipment and components	29
Containers and packaging	Equipment and components	17
Electrical equipment and machinery	Equipment and components	50
Healthcare equipment and supplies	Equipment and components	7
Semiconductors and semiconductor equipment	Equipment and components	8
Technology hardware and equipment	Equipment and components	36
Tires	Equipment and components	4
Banks, diverse financials, insurance	Other services	9
Education services	Other services	2
Healthcare providers and services and healthcare technology	Other services	1
Hotels, restaurants and leisure, and tourism services	Other services	23
Media	Other services	18
Pharmaceuticals, biotechnology, and life	Other services	31
Software and services	Other services	44
Specialized consumer services	Other services	1
Specialized financial services, consumer finance, insurance brokerage firms	Other services	2
Telecommunication services	Other services	36
Trading companies and distributors and commercial services and supplies	Other services	15
Solid waste management utilities	Other utilities	5
Water utilities	Other utilities	5
Electric utilities and independent power producers and energy traders (including fossil, alternative, and nuclear energy)	Power	37
Electric utilities and IPPs and energy traders	Power	1
Professional services	Professional services	118
Food production: agricultural production	Raw materials	12
Food production: animal source food production	Raw materials	7
Forest and paper products: forestry, timber, pulp, and paper, rubber	Raw materials	13
Mining: iron, aluminum, other metals	Raw materials	9
Mining: other (rare minerals, precious metals, and gems)	Raw materials	2
Real estate	Real estate	68
Food and staple retailing	Retailing	21
Retailing	Retailing	47
Air freight transportation and logistics	Transport	19
Air transportation: airport services	Transport	2
Ground transportation: highways and railtracks	Transport	3
Ground transportation: railroads transportation	Transport	15
Ground transportation: trucking transportation	Transport	10
Water transportation: ports and services	Transport	2
Water transportation: water transportation	Transport	3

## Footnotes

1 See Andersen et al. [75] for a definition of SBTs applicable beyond the translation of the Paris temperature goal to companies.

2 Scope 1 GHG emissions refer to direct emissions by a company and commonly relate to fuel combustion, physical or chemical processing, and leakages [76]. Scope 2 emissions refer to indirect GHG emissions from a company’s consumption of purchased electricity, steam, and heating/cooling [76].

3 Scope 3 GHG emissions refer to all indirect emissions not covered under scope 2, for example, related to production and transportation of purchased goods and services; processing and use of sold products; and employee commuting and business travel [76].

4 According SBTi: “Such procurement targets are acceptable if they are in line with procuring 80% of electricity from renewable sources by 2025 and 100% by 2030. Companies that already source electricity at or above these thresholds shall maintain or increase their share of renewable electricity” [27].

5 SBTi’s website [37] lists only the most recent date of target approval for a given company, hence ignoring if a company had SBT(s) approved an earlier date, followed by later approval of revised SBT(s). Data on SBT revisions is not readily available, but SBTi’s assessment [36] (page 15) indicates a substantial growth in the number of targets approved annually over the 2015–2020 period, consistent with the pattern of Fig. 1.

6 SBTi states that “Companies that fail to have their science-based target(s) validated and published within a 24-month period after commitment will be removed from the SBTi Companies Taking Action page and from all other partners’ websites” [27]. As of November 2021, however, SBTi’s website [37] listed 136 companies that announced commitments between 2015 and 2019 but still do not have an approved SBT. It is unclear if additional companies were, instead, removed from this list for failing to translate commitments into approved SBTs within the 24-month deadline.

7 According to SBTi, corporate net-zero emissions satisfy two criteria: “Reducing scope 1, 2, and 3 emissions to zero or to a residual level that is consistent with reaching net-zero emissions at the global or sector level in eligible 1.5 °C-aligned pathways” and “neutralizing any residual emissions at the net-zero target year and any GHG emissions released into the atmosphere thereafter” [42].

8 We used the search string “climate OR emission OR greenhouse ‘science based target*’,” filtering for 2015 and later. This yielded 440 results on May, 25, 2021.

9 We used the search string “TOPIC**:** (“science-based target*”) AND TOPIC**:** (climate OR emission OR greenhouse).” This yielded 20 results on May, 25, 2021.

10 Due to the emergent nature of SBT research, we decided to include preprints, i.e., full drafts of research papers shared publicly before undergoing peer review.

11 The MSCI (formerly Morgan Stanley Capital International) world index is a market capitalization-weighted index that comprises stocks from companies across the world and is used to benchmark the performance of global equity funds.

12 Bolton and Kacperczyk [52] acknowledge that lower significance is likely in part due to the smaller sample size of companies with SBTs than companies with internal targets.

13 We note that some companies incorrectly claim to have an approved SBT in their CDP disclosure and may therefore be part of the company sample in Freiberg et al. [53].

14 The shares of companies behind, ahead, or that already achieved SBTs are not reported directly in SBTi [36]. We estimated them from the company-level analysis presented in the study’s Appendix.

15 This can most likely be explained from SBTi [36]’s more restrictive inclusion criteria, such as not considering SMEs, only considering companies that reported to CDP and only considering “active” targets (for example, excluding companies that had already achieved their target but that had not set a new target). The differences in sampling approach also means that the two studies in some cases cover different SBTs for the same company.

16 SBTi [36] did not directly report on differences in target progress across emissions scopes. Based on the company-level analysis presented in the study’s Appendix, we found that companies were behind on 52% of scope 3 targets, while only behind on 34% of the targets that involve scope 1 and/or 2 (and behind on 38% of targets that involve all three emission scopes).

17 An emission imbalance occurs if all companies use the same SBT method and their SBTs do not add up to the global allowed emissions. Bjørn et al. [49] showed that all SBT methods involve some level of emission imbalance, due to mathematical approximations in target equations, and that the sign (i.e., whether there is an emission overshoot or “undershoot”) and size of the imbalance differ across methods.

18 Dagnet et al. [50] report that Walmart’s SBT for scope 3 made Pakistani supplier Yunus Textile Mills commit to setting its own SBT, as also reported in a 2017 blogpost by SBTi [77]. However, Yunus Textile Mills appear to have not developed a target, as it was not in SBTi’s target database, as of 14 December 2021 [37].

19 For example, SBTi encouraged companies to advocate in favor of the climate components of the Biden administration’s Build Back Better Act in its 2021 December newsletter.
